# A Double-Blind, Randomised, Crossover Trial of Two Botulinum Toxin Type A in Patients with Spasticity

**DOI:** 10.1371/journal.pone.0056479

**Published:** 2013-02-28

**Authors:** Fábio Coelho Guarany, Paulo Dornelles Picon, Nicole Ruas Guarany, Antonio Cardoso dos Santos, Bianca Paula Mentz Chiella, Carolina Rocha Barone, Lúcia Costa Cabral Fendt, Pedro Schestatsky

**Affiliations:** 1 Physical Medicine and Rehabilitation Service, Hospital de Clínicas de Porto Alegre (HCPA), Porto Alegre, Rio Grande do Sul, Brazil; 2 Graduate Program in Medical Sciences, Universidade Federal do Rio Grande do Sul (UFRGS), Porto Alegre, Rio Grande do Sul, Brazil; 3 Department of Internal Medicine, Hospital de Clínicas de Porto Alegre (HCPA), Porto Alegre, Rio Grande do Sul, Brazil; 4 School of Occupational Therapy, Universidade Federal de Pelotas (UFPel), Pelotas, Rio Grande do Sul, Brazil; 5 Pharmacy Department, Hospital de Clínicas de Porto Alegre (HCPA), Porto Alegre, Rio Grande do Sul, Brazil; 6 Universidade Federal do Rio Grande do Sul (UFRGS), Porto Alegre, Rio Grande do Sul, Brazil; 7 Neurology Service, EMG Unit, Hospital de Clínicas de Porto Alegre (HCPA), Porto Alegre, Rio Grande do Sul, Brazil; University of Pittsburgh, United States of America

## Abstract

**Background:**

Botulinum toxin type A (btxA) is one of the main treatment choices for patients with spasticity. Prosigne® a new released botulinum toxin serotype A may have the same effectiveness as Botox® in focal dystonia. However, there are no randomized clinical trials comparing these formulations in spasticity treatment. The aim of our study was to compare the efficacy and safety of Prosigne® with Botox® in the treatment of spasticity.

**Methodology/Principal Findings:**

We performed a double-blind, randomized, crossover study consisting of 57 patients with clinically meaningful spasticity. The patients were assessed at baseline, 4 and 12 weeks after Prosigne® or Botox® administration. The main outcomes were changes in the patients’ Modified Ashworth Scale (MAS), Functional Independence Measure (FIM) and Pediatric Evaluation of Disability Inventory (PEDI) scores and adverse effects related to the botulinum toxin. Both of the toxins were significantly effective in relieving the level of spasticity in adults and children. There were no significant differences found between the Prosigne® and Botox® treatments regarding their MAS, FIM and PEDI scores. Likewise, the incidence of adverse effects was similar between the two groups.

**Conclusion:**

Our results suggest that Prosigne® and Botox® are both efficient and comparable with respect to their efficacy and safety for the three month treatment of spasticity.

**Trial Registration:**

ClinicalTrials.gov NCT00819065.

## Introduction

Spasticity is a motor disorder characterized by a velocity-dependent increase in tonic stretch reflex [1] that might cause pain and disability [2], [3]. Together with motor rehabilitation, botulinum toxin type A (btxA) is considered one of the main treatment choices for patients with spasticity irrespective the causes [3]. Although there is no demonstrated functional improvement, the treatment of spasticity with botulinum toxin is justified by the possibility of pain and joint deformities relief, as well as by facilitating self-care such as dressing and bathing [4], [5]. Botulinum toxin formulations of the same serotype might present different efficacy and safety profile [6]. A recently released btxA, Prosigne®, has been used to treat spasticity. However, until now, there has been a lack of randomized, controlled trials reported in the literature that analyze the role of this drug in patients with increased muscle tone. Nevertheless two randomized clinical trials demonstrated similar effectiveness comparing Prosigne® and Botox® in focal dystonia [7], [8]. In this non-inferiority study, we analyzed the efficacy and safety of Prosigne® compared to Botox® in patients with spasticity resultant from several causes.

## Methods

The protocol for this trial and supporting CONSORT checklist are available as supporting information; see Checklist S1 and Protocol S1.

A double-blind crossover trial was performed as recorded in the protocol http://clinicaltrials.gov/(register number: NCT 00819065). The study shows the same protocol that was approved by the Research Ethics Committee (institutional review board-equivalent) of the Hospital de Clínicas de Porto Alegre. All patients provided written informed consent for their participation in the study. Written informed consent was obtained from the next of kin, caretakers, or guardians on the behalf of the minors/children participants involved in this study. The patients were consecutively recruited from our spasticity disorders clinic (Hospital de Clínicas de Porto Alegre, Brazil) that met the selection criteria were enrolled in a consecutive sequence allocation (1 to 60). A permuted blocks randomization was made by the only unblinded pharmacist using the web site www.randomization.com and only the pharmacist has access to the generated list during all the study period the pharmacist was responsible for deliver the study medication outside the pharmacy department, none of the blinding staff (investigators, patients, caregivers) had access to the pharmacy department. Special labels were fixed on the application syringe for each treatment ensuring the blinding conditions. The blinding condition was broken only after all data was valid and completed for statistical analysis. The rehabilitation program was also offered to all of the patients on a regular basis. Adverse events were measured using a semi-structured questionnaire.

### Study Design: Crossover

All patients were randomized to receive either Prosigne® (Lanzhou Biological Products Institute) or Botox® (Allergan Pharmaceuticals). After 12 weeks, those who received Prosigne® switched to Botox®, and vice-versa (crossover point). Patients were followed for 12 more weeks when the study was concluded. The clinical results from both phases (pre- and post crossover) were merged and compared according to different botulinum toxin formulations (Prosigne® versus Botox®).

### Inclusion and Exclusion Criteria

**Figure 1:**
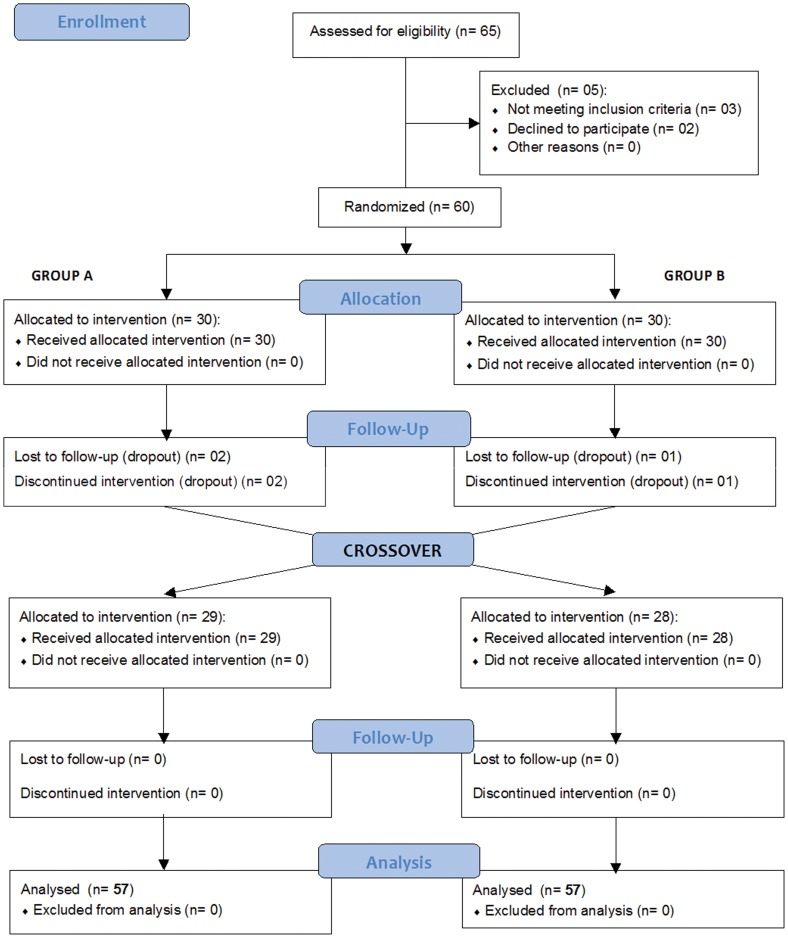
CONSORT 2012 Flow diagram of patients.

Patients with any cause of spasticity were eligible to participate in the study if they were older than 2 years and had no previous btxA treatment or had gone without treatment within the last 6 months. We excluded all of the patients that exhibited fixed contracture (MAS = 4) or profound atrophy in the affected limb, were currently undergoing surgical treatment for spasticity, used agents that affected neuromuscular transmission or had known contraindications to btxA, or those patients who were currently pregnant. Figure 1 shows the flow diagram of patients in the trial.

### Treatment, Dose Regimen, and Titration

The patients were randomized to Botox® at the usual effective dose or to Prosigne®, at a ratio of 1∶1 (1 Botox® unit to 1 Prosigne® unit). This ratio was chosen based on doses reported in previous studies [7], [8]. Both btxAs were diluted with sterile sodium chloride (10 U/0.2 mL). The dose of btxA per injection was defined according to the universally accepted guidelines [9]. The btxA was prepared at each injection administration by a pharmacist (B.P.M.C.) blinded to the patient’s identification. The sites of injection, number of injections and dose at each treatment session were consistent between both treatments. The outcome measurements of the patients were evaluated in the baseline, 4 and 12 weeks after the first and second injection. The principal investigator (F.C.G.), who has extensive experience in treating spastic patients, rated each patient at each visit. At 12 weeks of the first injection (Prosigne® or Botox®) the treatments were changed (Prosigne® for Botox®, and *vice-versa*) still maintaining the double-blind fashion. The appointment for re-injection was pre-defined at baseline and maintained until the end of the study. The patients that received oral medications were kept on a stable dose for at least 30 days before entry and throughout the study. There were no changes to methods or in the trial outcomes after trial commencement.

The main outcomes in this study were as follows: 1) the spasticity level, as measured by the Modified Ashworth Scale (MAS), subjectively measured improvement from 0 to 4; 2) the functionality level, as measured by the Functional Independence Measure (FIM) scale for patients older than 8 years, ranged from 18 to 126; and 3) the PEDI scale for patients younger than 8 years old ranged from 0 to 100. The adverse effects were assessed via a structured clinical interview with open questions.

### Sample Size and Data Analysis

The sample size was calculated using PEPI (Version 2.0) using a significance of 0.05. Assuming that only a difference of ±0.7 points in the main outcome (mean MAS score changes) would be clinically relevant [10], and providing a power of 95% for comparisons, it was estimated that a total number of 56 patients would be necessary for a non-inferiority trial. The data distribution was assessed using the Shapiro-Wilk that showed a normal distribution for all outcome variables. Therefore, we used Student t test and ANOVA for repetitive measures with generalized estimating equations and Bonferronís post-hoc test, when needed.

The MAS score was obtained from all of the muscles involved in the spasticity of the entire group of patients and express it as mean changes at each visit. We considered the baseline, 4-week (peak effect) and 12-week results of MAS score for analysis. Because of the crossover design, to evaluate the influence of the carry-over effect from one drug to the other, we also analyzed differences of changes in the MAS, FIM and PEDI scores at the 4th week before and after the crossover using the Student´s t test for each comparison. For the adverse events analysis, we first divided the patients into 2 groups (those who received more and those who received less than 10 U of botulinum toxin per kilogram) and used the McNemar chi-square test. We also analyzed the dose-dependent adverse events using the chi-square test with the Yates correction. We used a *p* value of 0.05 for statistical significance. However, because a more informative approach is usually preferred in non-inferiority and equivalence trials we expressed results significance using confidence intervals for the main outcomes [11].

## Results

Initially, a total of 60 patients were randomized to the btxA treatments. There were 3 withdrawals (2 from the Prosigne® and 1 from the Botox® group) after the first btxA injections due to unknown reasons. Thus, 57 patients were completely assessed in each intervention of this trial (see Figure 1).

Most of our patients had cerebral palsy (65%) for less than 12 years (56%). The demographic and clinical characteristics of the patients are summarized in Table 1. The mean btxA dose per/kg administered to the patients under and above 12 years old were 7.8±3.8 U and 3.3±1.5 U (t-test; *p*<0.001), respectively. Table 2 shows the mean dose of btxA for each muscle.

**Table 1:** Demographic and clinical characteristics of patients (n = 57).

Demographic data	n (SD)
♂	26
♀	31
Children (2–11 y)	32
Adults (≥12 y)	25
MAS	2.07 (0.5)
FIM	101.6 (21.1)
PEDI	53.08 (10.5)
Weight (kg)	40.9 (25)
Height (cm)	135 (27)
BMI	20.4 (6.3)
Cerebral palsy	38
Stroke	16
Other	3

MAS: Modified Ashworth Scale; FIM: Functional Independence Measure; PEDI: Pediatric Evaluation of Disability Inventory; BMI: Body Mass Index; SD, Standard Deviation.

**Table 2:** Muscles and btxA doses.

Muscle	Mean Doses U* (min–max)
*Pectoralis major*	72 (30–100)
*Biceps brachii*	71 (20–100)
*Brachioradialis*	38 (30–60)
*Flexor carpi radialis*	32 (10–60)
*Flexor carpi ulnaris*	33 (10–60)
*Pronator quadratus*	19 (10–30)
*Pronator teres*	19 (10–40)
*Flexor digitorum profundus*	24 (10–40)
*Flexor digitorum superficialis*	24 (10–40)
*Flexor pollicis longus*	15 (10–20)
*Adductor pollicis*	10 (10–10)
*Opponens pollicis*	10 (10–10)
*Adductor magnus*	53 (30–100)
*Rectus femoris*	25 (25–25)
*Semitendinosus*	33 (20–50)
*Semimembranosus*	33 (20–50)
*Tibialis posterior*	35 (20–50)
*Gastrocnemius*	64 (20–100)
*Soleus*	26 (10–50)

* Units btxA.

After 4 weeks of each injection (peak effect), the combined data from the two phases of the study (before and after crossover) showed that both Prosigne® and Botox® treatment were significantly effective in reducing the MAS scores; however, we found no detectable differences between the two drugs (t-test; Effect Size (ES): −0.31; 95% CI: −0.68 to 0.06). After 12 weeks (at the end of the study), significant differences were again observed between the baseline MAS scores for both of the drugs, and no differences were found between the Prosigne® and Botox® treatments (t-test; ES: −0.13; 95% CI: −0.49 to 0.24). The results of all clinical scores after botulinum toxin treatment are summarized in Table 3.

**Table 3:** End-points for clinical outcomes at baseline, 4 weeks and 12 weeks after botulinum toxin treatment.

Scale	Subscale	Time point	Prosigne® (n = 57) Mean (SD)	Botox® (n = 57) Mean (SD)	Between-group difference in end-point (95% CI)	Effect size (95% CI)
MAS						
		Baseline	1.93 (0.5)	1.84 (0.46)	0.09 (−0.06 to 0.23)	0.18 (−0.19 to 0.54)
		4 weeks	1.28 (0.41)	1.42 (0.48)	−0.14 (−0.26 to −0.01)	−0.31 (−0.68 to 0.06)
		12 weeks	1.52 (0.39)	1.59 (0.53)	−0.07 (−0.21 to 0.07)	−0.13 (−0.49 to 0.24)
FIM						
		Baseline	103.20 (23)	102.57 (23.67)	0.63 (−1.43 to 2.68)	0.03 (−0.65 to 0.7)
		4 weeks	102.2 (29.5)	105.5 (16.2)	−3.3 (−2.1 to 4.5)	−0.14 (−0.76 to 0.48)
		12 weeks	103.92 (22.85)	103.07 (23.36)	0.85 (−1.39 to 3.09)	0.04 (−0.64 to 0.71)
PEDI						
	Self-care					
		Baseline	56.86 (12.89)	58.40 (13.31)	−1.54 (−5.09 to 2)	−0.12 (−0.56 to 0.32)
		4 weeks	58.9 (14.3)	62.4 (12.1)	−3.5 (−2.9 to 1.2)	−0.26 (−1.21 to 0.73)
		12 weeks	61.30 (13.55)	61.38 (13.74)	−0.08 (−2.60 to 2.43)	−0.01 (−0.44 to 0.43)
	Mobility					
		Baseline	49.17 (15.07)	51.75 (14.29)	−2.58 (−5.19 to 0.02)	−0.18 (−0.61 to 0.27)
		4 weeks	51.8 (16.9)	52.3 (8.5)	−0.5 (−0.8 to 1.9)	−0.04 (−1.00 to 0.93)
		12 weeks	53.60 (14.07)	51.61 (14.52)	1.98 (0.42 to 3.55)	0.14 (−0.30 to 0.58)
	Social Function					
		Baseline	57.93 (11.72)	57.14 (10.74)	0.78 (−2.34 to 3.91)	0.07 (−0.37 to 0.51)
		4 weeks	58.1 (11.3)	61.5 (10.7)	−3.4 (−3.7 to 0.5)	−0.31 (−1.26 to 0.68)
		12 weeks	61.14 (12.55)	61.93 (11.43)	−0.78 (−3.51 to 1.94)	−0.07 (−0.50 to 0.37)

MAS: Modified Ashworth Scale; FIM: Functional Independence Measure; PEDI: Pediatric Evaluation of Disability Inventory; SD: Standard Deviation; CI: Confidence Interval.

We also performed a subgroup analysis of the children and adults separately, and similar results were obtained for the changes observed in the MAS scores. When comparing the effect of Prosigne® or Botox® after 4 weeks in both phases of the study, no differences were detected (*p* = 0.3, before crossover; *p* = 1.0, after crossover). Regarding functional changes, no significant changes were observed in the FIM or PEDI scores, from the baseline to 4 or 12 weeks, for any of the treatments (Table 3).

The two most common adverse effects, which were reported by patients in both groups, were local pain and skin erythema, both of which were transient and well tolerated. In addition, the incidence of adverse effects was similar between the groups (Fisher exact test; *p*>0.5 for all of the events), as shown in Table 4. In addition, patients who were less than 12 years old did not exhibit an increased chance of adverse effects with either Botox® (χ^2^ = 0.016; *p* = 0.9) or Prosigne® (χ^2^ = 0.001; *p* = 0.9) treatment. When present, the adverse effects were not dose-dependent (χ^2^ = 0.009; *p* = 0.9 for Prosigne® and χ^2^ = 0.54; *p* = 0.5 for Botox®). In the subgroup analysis, the age of the patients (children and adults) had no effect on the incidence of adverse effects irrespective of the drug treatment. When we compared only btxA systemic side effects (dry mouth, somnolence, fatigue, etc.) between groups, the absolute number of events were 15 for Prosigne® and 7 for Botox®. However, this difference did not reach statistical significance (Fisher exact test *p* = 0.25).

**Table 4:** Number of patients (total = 57) with clinical adverse effects (chi-square test with Yates correction).

Adverse events	n	Prosigne®	Botox®	*p*
Local pain	9	5	4	0.7
Skin erythema	5	1	4	0.2
Somnolence	3	2	1	0.6
Local ecchymosis	3	1	2	0.6
Muscle weakness	3	2	1	0.6
Tearing	2	1	1	0.9
Cough	1	0	1	0.5
Fever	1	0	1	0.5
Pruritus	1	0	1	0.5
Inappetence	1	1	0	0.5
Blurred vision	1	0	1	0.5
Vomit	1	1	0	0.5
Red eye	1	1	0	0.5
Oedema	1	1	0	0.5
Nausea	1	1	0	0.5
Polydipsy	1	1	0	0.5
Dry mouth	1	1	0	0.5
Chest pain	1	1	0	0.5
Hypertension	1	1	0	0.5
Fatigue	1	1	0	0.5

## Discussion

To our knowledge, this is the first randomized, controlled trial that compares Prosigne® with Botox® for the treatment of spasticity. The results of this study showed that both drugs are equally effective and safe in patients with spasticity. This finding is consistent with recent trials in patients with focal dystonia [7], [8]. As expected the muscular tonus reduction was not associated with significant functional improvement with either type of botulinum toxin. This is consistent with other studies that did not observe significant functional gain with this treatment in patients with spasticity [2]–[4], [12]–[14].

Regarding the MAS (main outcome), its confidence interval very nearly included the 0.7 (minimal clinically significantly difference) and, therefore, it could be possible that a superiority of Prosigne® would be appreciated if the sample size was larger. Therefore, further studies with higher number of patients are justified in the future to clarify this point.

The presence of adverse effects (such as dry mouth, somnolence and fatigue) may be due to the systemic spread of the toxin outside of the muscle injection site and, because there effects were not dependent on the btxA dose, nor the patient’s age, they should be considered idiosyncratic. Although not statistically significant, the adverse events seemed to be more frequent in patients treated with Prosigne®, as seen in Table 4. Therefore, these findings need to be further addressed in future studies of Prosigne® using a higher number of patients.

In some developing countries such as Brazil, the government provides free distribution of botulinum toxin to patients who fulfill the clinical criteria according to evidence-based guidelines [13]. Therefore, the reduced cost of Prosigne® may promote price competition with distinct drug formulations and amplify the availability of the drug to a larger number of patients. Indeed, this has already occurred for patients with focal dystonia in some parts of Brazil [14].

Our study has some limitations. First, due to ethical reasons, we did not perform a washout period and, considering the half-life of 12 weeks, this might have induced a carry-over effect of one drug upon the other. However, the magnitude of an eventual carry-over effect was not statistically different between groups right before the crossover (12^th^ week). Second, we did not follow the patients for a longer period of time, but there is good evidence that 4 weeks is an optimal time point for the assessment of the treatment efficacy of botulinum toxin on spasticity [12].

In conclusion, despite the limitations of this study, our results suggest that Prosigne® and Botox® are equally effective and safe after 12 weeks for the treatment of spasticity. Because btxA is considered to be a high-cost treatment, our findings may be of interest from a pharmacoeconomic perspective, especially in developing countries. Future studies should further explore the comparability of different btxA formulations and serotypes, using a larger sample size and with additional specifications, including safety, cost-effectiveness and cost-utility parameters.

## Supporting Information

Protocol S1 Randomized Double-blind Clinical Trial Comparing Two Commercial Formulations of Botulinum Toxin Type A in the Treatment of Spasticity.
(DOC)

Checklist S1 CONSORT 2010 checklist of information to include when reporting a randomised trial.
(DOC)
