# Supplementary material for: A Double-Blind, Randomised, Crossover Trial of Two Botulinum Toxin Type A in Patients with Spasticity
Source: PLoS One. 2013 Feb 28;8(2):e56479. doi: 10.1371/journal.pone.0056479 (PMC3585303; doi:10.1371/journal.pone.0056479)
Supplement: Protocol S1 — Randomized Double-blind Clinical Trial Comparing Two Commercial Formulations of Botulinum Toxin Type A in the Treatment of Spasticity. (DOC) [file pone.0056479.s001.doc]

**Protocol S1. Randomized Double-blind Clinical Trial Comparing Two Commercial Formulations of Botulinum Toxin Type A in the Treatment of Spasticity**

Botulinum toxin type A has been used clinically in spasticity treatment for over a decade. There are in Brazil, nowadays, three commercial formulations of BTA, wich, though the same type of toxin, are different biological products that differ about storage, dilution and dosage. The units of one toxin are exclusive of that product, and an international standard-unit lacks. Each kind of BTA has been through validation and safety studies, but there are very few comparative studies between them. Thus, the purpose of this study is to compare the efficacy between two commercial formulations of BTA in the treatment of spasticity. We will also evaluate the safety, adverse effects and cost of each formulation.

Will be included in this study patients with Spasticity by IC10 criteria, 2 years and older, both genders, currently in treatment BTA (at least six months without the use) and/or with indication for BTA treatment for spasticity, who must not have any contraindications for the drug, at the Spasticity outpatient clinic of the Hospital de Clínicas de Porto Alegre. We will enroll patients on current treatment for spasticity at the Spasticity outpatient clinic of HCPA, already in treatment with BTA or starting that treatment based on their doctors' decision, who are willing to participate in the trial by signing an informed consent form. All patients included must agree to participate in the study by signing an informed consent form.

Patients will be randomized into two groups of 28 individuals each (total of 56 patients) and will receive BTA from laboratory Allergan and Lanzhou, one at time of allocation and the other three months after, in a crossover model.

These drugs are both approved by NATIONAL HEALTH SURVEILLANCE AGENCY (ANVISA) for the treatment of spasticity and provided by the national public health system (SUS). Application will be performed by a trained investigator, unaware of the kind of BTA in use. Standard dilutions of both toxins will be employed, within the usual dose and application spots (for patients already in treatment) or standard (for patients yet to start treatment). Identical dose will be used, with 2ml of saline solution 0.9% dilution for each 100units, thus, there will be no difference for the patient or investigator concerning the applied volume, assuring appropriate masking.

Follow-up visits will be performed after four, twelve, sixteen and twenty-four weeks, by investigator unaware of the kind of BTA in use. The modified Ashworth Scale will be applied at every visit by three medical investigators isolatedly, duly trained and blinded to the intervention. Life quality will be assessed at time of allocation, four and twelve weeks after, using WHOQOL-Bref, YQOL-R and Children's Life Quality Assessment Questionnaire, respecting the age of the patient. Functional capability will also be evaluated, through the Functional Independence Measure Scale (FIM) for adults and PEDI scale for children, at the same time frames as the life quality assessment instruments.

The primary end-point consists on the maximum degree of effect assessed through passive measurement of muscular tonus and quantified by the modified Ashworth Scale. Secondary end-points are presence, kind and duration of adverse effects of each treatment and perception of improvement of symptoms by the patient himself or caregiver.

Statistical analysis will be performed using the SPSS package for Windows and descriptive analysis will be provided by absolute and relative frequencies and mean±standard deviation for quantitative variables and percentages for qualitative ones. The T of Student or Mann-Whitney tests will be used for independent samples, and chi-square and Fischer's exact tests will be performed when necessary. Significance level will be 5%.There will be no additional risk for the patients, since the drugs involved in this trial have already been tested individually for efficacy and safety and are currently being used in medical practice for the treatment of these conditions.

The Public Health State Secretariat will be responsible for providing the drugs, according to routine pharmaceutical assistance, and there will be no additional cost for the hospital nor health system.
